# Aspergillus endocarditis: Diagnostic criteria and predictors of outcome, A retrospective cohort study

**DOI:** 10.1371/journal.pone.0201459

**Published:** 2018-08-09

**Authors:** Marwa Sayed Meshaal, Dina Labib, Karim Said, Mohammed Hosny, Mohammed Hassan, Said Abd Al Aziz, Amani Elkholy, Mervat Anani, Hussien Rizk

**Affiliations:** 1 Cardiovascular Medicine Department, Kasr Al-Ainy Teaching Hospital, Cairo University, Cairo, Egypt; 2 Cardiothoracic Surgery Department, Kasr Al-Ainy Teaching Hospital, Cairo University, Cairo, Egypt; 3 Clinical Pathology Department, Kasr Al-Ainy Teaching Hospital, Cairo University, Cairo, Egypt; IRCCS Policlinico S.Donato, ITALY

## Abstract

**Background:**

Fungal Endocarditis (FE), a relatively rare disease, has a high rate of mortality and is associated with multiple morbidities. *Aspergillus* endocarditis (AE) is severe form of FE. Incidence of AE has increased and is expected to rise due to an increased frequency of invasive procedures, cardiac devices and prosthetic valves together with increased use of immune system suppressors. AE lacks most of the clinical criteria used to diagnose infective endocarditis (IE), where blood culture is almost always negative, and fever may be absent. Diagnosis is usually late and in many cases is made post-mortem. Late or mistaken diagnosis of AE contribute to delayed and incorrect management of patients. In the current study we aimed to describe the clinical, laboratory and imaging characteristics of AE, to identify predictors of early diagnosis of this serious infection.

**Methods:**

Patients with definite/possible IE, as diagnosed by the Kasr Al-Ainy IE Working Group from February 2005 through June 2016, were reviewed in this study. We compared the demographic, clinical, laboratory and imaging criteria of AE patients to non-fungal IE patients.

**Results:**

This study included 374 patients with IE in which FE accounted for 43 cases. *Aspergillus* was the most common fungus (31 patients; 8.3%) in the patient group. Lack of fever and acute limb ischemia at presentation were significantly associated with AE (p < 0.001, p = 0.014, respectively). Health care associated endocarditis (HAE) and prosthetic valve endocarditis (PVE) were the only significant risk factors associated with AE (p < 0.001 for each). Mitral, non-valvular, and aortotomy site vegetations, as well as aortic abscess/pseudoaneurysm, were significantly associated with AE (p = 0.022, p = 0.004, p < 0.001, and p < 0.001, respectively). Through multivariate regression analysis, HAE, PVE, aortic abscess/pseudoaneurysm, and lack of fever were strongly linked to AE. The probability of an IE patient having AE with HAE, PVE, and aortic abscess/pseudoaneurysm, but no fever, was 0.92. In contrast, the probability of an IE patient having AE with fever, native valve IE, but no health-care associated IE and no abscess/pseudoaneurysm, was 0.003. Severe sepsis and mortality in the Aspergillus group were higher as compared to the non-fungal group (p = 0.098 and 0.097, respectively). Thirteen AE patients died during hospitalization. PVE, the use of single versus dual antifungal agents, severe heart failure, and severe sepsis were significant predictors of mortality (p = 0.008, 0.012, 0.003, and 0.01, respectively).

**Conclusion:**

To our knowledge, this is the first study to address diagnostic criteria for AE. Through multivariate regression analysis, absence of fever, HAE, PVE, and aortic abscess/pseudoaneurysm were strong predictors of AE. Use of these criteria my lead to earlier diagnoses of AE. Early treatment of AE patients with voriconazole in combination with other antifungal agents may be possible based on the previously mentioned criteria, which may facilitate better patient outcomes.

## Introduction

Although fungal endocarditis (FE) was considered to be an uncommon disease, occurrence of this disease is rising and is expected to continue to increase. Factors that contribute to this increasing rate of FE include higher number of patients subjected to invasive procedures, improved survival of patients with complex cardiac disease that mandates implantation of cardiac valves and devices, and the prolonged use of intravenous (IV) catheters and broad spectrum antibiotics [1]. FE affects nearly 0.1% of all prosthetic cardiac valves [2]. *Aspergillus* accounted for 24–28% of all FE cases and between 0.25%-2.5% of all infective endocarditis (IE) cases reported in English language indexed medical journals between 1965–1995 [1, 2]. However, *Aspergillus* endocarditis (AE) still lacks specific criteria for early diagnosis, a challenge that is exacerbated by the fact that *Aspergillus* blood culture is always negative [3, 4] and therefore cannot be used for diagnostic purposes. In recent studies, AE was diagnosed post-mortem in approximately one-third of cases and was diagnosed preoperatively in less than half of patients [5]. FE is a disease associated with high rates of mortality and morbidity [3, 4]; these rate are even higher for AE [2, 5]. Cardiac surgery is commonly used in the treatment of AE, although mortality rates remain high [5]. Delayed diagnosis of AE due to lack of clinical and laboratory criteria may contribute to delayed treatment and ultimate poor outcomes. In this study, predictive factors for AE diagnosis were identified that may help with early diagnosis of AE, which would facilitate earlier treatment and better outcomes.

## Patients and methods

All enrolled patients diagnosed with definite/possible IE by the Kasr Al-Ainy IE Working Group in the period from February 2005 through June 2016 were reviewed in this study. The IE Working Group used AHA/ACC guidelines and ESC guidelines for the diagnosis and management of IE patients [3, 4, 6, 7].

Per patient, prior to staring antibiotics, at least three sets of blood culture were collected from separate venipunctures, with the first and last samples drawn at least 1-hour apart. Each blood culture set consisted of one BACTEC Plus aerobic/F and one BACTEC Plus anaerobic/F culture vial (Becton Dickinson, Sparks, MD, UAE). For adult patients, approximately 10 ml of blood were collected per culture vial. For pediatric patients, the collected blood volume was adjusted according to body weight. Blood culture bottles were incubated in a BACTEC 9240 instrument for 14 days. Positive blood culture bottles were subcultured onto 5% Sheep blood agar, chocolate blood agar, MacConkey agar, and Sabouraud dextrose agar plates (Oxoid Ltd, UK). Microbial colonies were identified by Gram-stain, colony morphology, and the VITEK-2 system (BioMerieux).

Surgically excised materials including excised valves, vegetations, infected prosthesis, aortic abscess, and emboli, were submitted for Gram-staining, potassium hydroxide preparation (KOH), histopathology examination, and microbial culture on 5% Sheep blood agar, chocolate blood agar, MacConkey agar, and Sabouraud dextrose agar plates (Oxoid Ltd, UK). Organism identification and antibiotic susceptibility testing was performed on a VITEK-2 system (BioMerieux) [3, 4, 6, 7, 8].

Serodiagnosis for the detection of antibodies specific to endemic zoonotic agents (*Brucella*, *Bartonella*, and *Coxiella*) and Aspergillus Galactomannan Antigen was performed according to manufacturer’s instructions. Anti-*Brucella* antibodies were detected using the tube agglutination test (Linear Chemicals, Montgat-Barcelona, Spain). Testing for IgG antibodies against *Bartonella henselae* and *Bartonella quintana* and for IgG, IgM, and IgA antibodies against *Coxiella burnetii* was carried out using the indirect immunofluorescence assay (Vircell S.L. microbiologist, Granada, Spain). A patient was considered to have brucellosis when *Brucella* antibody titers were at least 1:320, Bartonella endocarditis when *Bartonella* IgG titers were at least 1:800, and Coxiella endocarditis when *Coxiella* phase I IgG titers were at least 1:800 [3, 4, 6, 7, 9].

The Platelia EIA kit (BioRad) was used to detect Aspergillus Galactomannan Antigen. According to kit instructions, patients with an index ≥ 0.5 are considered to be positive for Aspergillus Galactomannan Antigen. However, in this study an index of ≥1 was used as the threshold for diagnosis of Aspergillus endocarditis, which was based on the two-fold or greater index values observed in patients with positive culture results for *Aspergillus*. For a patient to be considered positive for the Aspergillus Galactomannan Antigen, the test had to be positive in two repeated tests performed within one week and the patient could not be taking any beta-lactam antibiotics.

Positive AE refers to either identification of Aspergillus spp. by direct microscopy with KOH preparation, positive culture from blood or surgically excised material, or histopathological examinations showing evidence of endocarditis and tissue invasion by branching filamentous septate hyphae and/or a positive test result for Aspergillus Galactomannan Antigen as described above [3, 4, 6, 7, 8]. Trans-thoracic echocardiography (TTE) was performed within 24 hours of admission. Transesophageal echocardiography (TEE) was performed, as indicated, within 48–72 hours. All images were standardized according to the guidelines of the American Society of Echocardiography [10, 11].

Health-care associated IE (HAE) was defined as:

1. Nosocomial IE: IE contracted ≥ 48 hours after hospital admission.
2. Non-nosocomial IE: IE appearing within: a) 1-month of receiving IV cannulation, chemotherapy, or dialysis; b) 3-months after admission into an acute care facility, or c) any time after admission into a nursing home.

As all authors were involved in care of enrolled patients and had access to their data, patient data were not aggregated, anonymized, or de-identified prior to access to and analysis.

### Statistical analysis

All data were analysed using the SPSS version 24 statistical software and R statistical package version 3.4.1, with two-tailed p-value < 0.05 indicating statistical significance. Normally distributed numerical values were reported as mean ± standard deviation (SD). For variables with a skewed distribution, data were expressed as median and inter-quartile range. Qualitative variables were presented as counts and percentages. Comparisons of continuous data between groups were made using the two-sample t test or the Wilcoxon rank sum test as appropriate. The Chi-square test or Fisher’s test were used to make between-group comparisons as appropriate. Stepwise multivariable logistic regression was performed to determine predictors of FE and mortality.

## Results

We enrolled a total of 374 patients with definite/possible IE between February 2005 and June 2016 in the study. Table 1 shows the causative microorganism identified from each patient in the database of IE Working Group. In 141 (37.7%) patients, the causative microorganism could not be detected by blood or tissue culture or serology (culture negative/serology negative cases). Of the entire patient population, 11.5% of the patients had FE, of which *Aspergillus* was the most common fungus (31/374 patients; 8.3%). Of the non-fungal group, *Staphylococcus* was the most common bacterium (24.1%), followed by *Streptococcus* (11.2%). Polymicrobial infection was detected in seven patients in both the fungal and bacterial IE groups (14/374; 3.7%).

**Table 1:** Causative microorganism.

Organism	Number (%)*
Bacterial	190 (50.8)
Staphylococci	90 (24.1)
Streptococci	42 (11.2)
Enterococci	15 (4)
Gram-negative	24 (6.4)
Zoonotic (Coxiella, Bartonella, Brucella)	22 (5.9)
Others	3 (0.8)
Fungal	43 (11.5)
Aspergillus	31 (8.3)
Candida	9 (2.4)
Mucormycosis	2 (0.5)
Penicillium	1 (0.3)
Unknown organism	141 (37.7)

*Sub-categories do not sum to 100% due to polymicrobial infection in some patients.

*Aspergillus* was diagnosed by tissue culture in 26 of the 31 AE patients and by the Aspergillus Galactomannan Antigen assay alone in the remaining five patients. These five patients died shortly before having cardiac surgery; autopsy was declined by the families. The Aspergillus Galactomannan Antigen was detected in an additional 18 AE patients. In eight AE patients Galactomannan Antigen result was missing.

### Characteristics of AE patients versus non-fungal group

The 31 AE patients were compared to the non-fungal group (bacterial IE and unknown microorganism) comprised of 331 patients. The clinical and echocardiographic characteristics of both groups are shown in Table 2. Both groups were relatively young (mean ages of 30.7 ± 16.43 years and 32.4 ± 12.01 years for the *Aspergillus* and non-fungal groups, respectively; p-value 0.55). Interestingly, fever was present in only 67.7% of the *Aspergillus* group as compared to 92.4% of the non-fungal group (p < 0.001). There was a significant association between AE and acute limb ischemia at presentation (p = 0.014).

**Table 2:** Clinical and echocardiographic characteristics of AE and non-fungal IE groups.

Variable	Aspergillus (n = 31)	Non-fungal (n = 331)	p-value
**Clinical characteristics**			
Age, years	30.7 ±16.43	32.4 ±12.01	0.548
Male gender	16 (51.6)	203 (61.3)	0.29
Symptom duration before referral, days	28 (57)	30 (66)	0.509
Fever	21 (67.7)	306 (92.4)	***< 0*.*001***
CVS* at presentation	3 (10)	58 (17.5)	0.292
Acute limb ischemia at presentation	5 (21.7)	19 (5.7)	***0*.*014***
**Predisposing factors**			
HAE	27 (87.1)	55 (16.6)	***< 0*.*001***
IV drug abuse	2 (6.5)	37 (11.2)	0.555
Dialysis	1 (3.2)	22 (6.6)	0.707
Malignancy	0	6 (1.8)	1.0
DM	1 (3.6)	18 (5.4)	1.0
Chronic steroid therapy / Collagen disease	1 (3.2)	21 (6.3)	0.708
Prior IE	0	13 (3.9)	0.614
**Underlying heart disease**			
Normal heart	5 (16.1)	69 (20.8)	0.533
Rheumatic heart disease	8 (25.8)	120 (36.3)	0.245
Congenital heart disease	2 (6.5)	29 (8.8)	1.0
Prosthetic valve	20 (64.5)	82 (24.8)	***< 0*.*001***
**Echocardiographic characteristics**			
Mitral vegetations	10 (32.3)	178 (53.8)	***0*.*022***
Aortic vegetations	13 (41.9)	115 (34.7)	0.423
Right-sided IE	3 (9.7)	63 (19)	0.197
Non-valvular vegetations	6 (19.4)	14 (4.2)	***0*.*004***
Aortotomy site vegetations	11(33.3)	0	***< 0*.*001***
Aortic abscess / pseudoaneurysm	13 (43.3)	36 (10.9)	***< 0*.*001***
TTE diagnostic	12 (52.2)	203 (61.3)	0.385
EF %	58.4 ± 9.02	61.2 ± 10.48	0.215

Data are presented as mean ± SD, n (column %), or median (interquartile range).

*CVS, cerebro-vascular accident.

Through univariate analysis, HAE and prosthetic valve IE (PVE) were the only significant risk factors associated with AE (p < 0.001 for each). HAE was present in 27 patients (87.1%) of the AE group. The most frequent culprit procedure was cardiac surgery (23 patients; 74.2% of the *Aspergillus* group). Dialysis, gynecology surgery, and hospitalization/IV line were the culprit in four patients. One patient was an IV drug abuser, one patient had 1ry aspergillus lung focus, while three patients had no known portal of entry.

**Fig 1:**
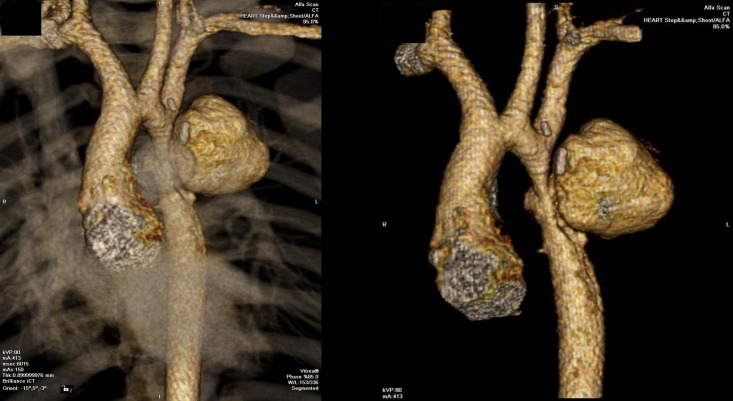
Multi-slice CT aortography showing an aortic pseudoaneurysm at the site of surgical coarctation repair in a 6-year old child.

**Fig 2:**
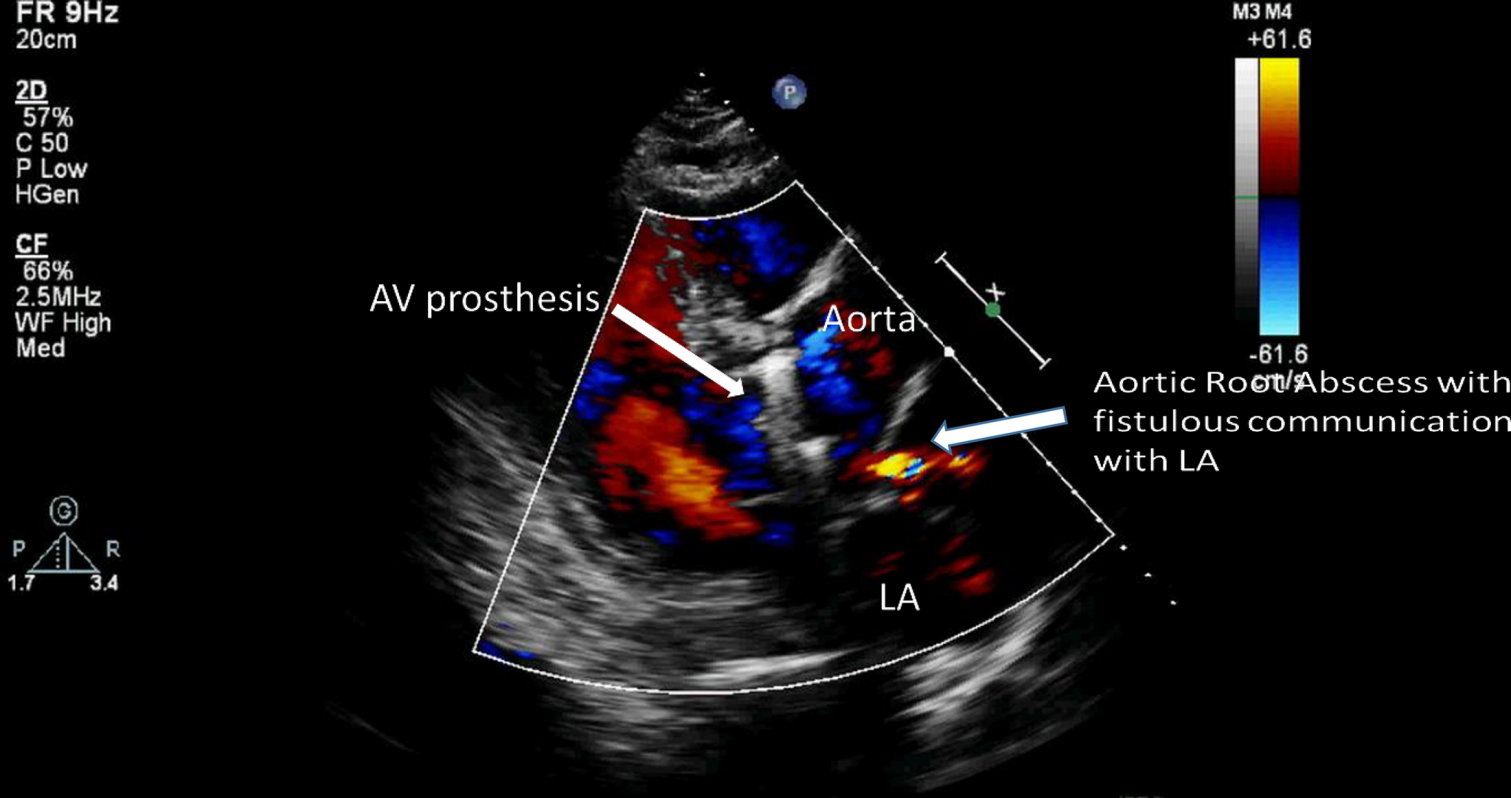
Transthoracic echocardiography showing a metallic aortic prosthesis with an aortic root abscess complicated by a fistula to the left atrium.

Moreover, mitral, non-valvular, and aortotomy site vegetations, as well as aortic abscess/pseudoaneurysm, were significantly associated with AE (p = 0.022, p = 0.004, p < 0.001, and p < 0.001, respectively). Examples of the diagnostic imaging modalities in patients are shown in Figs 1 and 2.

Significant univariate predictors of AE were entered in a stepwise logistic regression model with backward elimination. The details of the constructed regression model are shown in Table 3. HAE was the most powerful predictor of AE (p < 0.001), followed by absence of fever (p = 0.006), aortic abscess/pseudoaneurysm (p = 0.009), and PVE (p = 0.017). The probability of an IE patient having AE accompanied by HAE, PVE, and aortic abscess/pseudoaneurysm, but without fever, is 0.92. In contrast, the probability of an IE patient having AE accompanied by fever and native valve IE, but no HAE and no abscess/pseudoaneurysm, is 0.003. The sensitivity, specificity, positive predictive value (PPV), negative predictive value (NPV), and overall diagnostic accuracy of predicting AE based on these four variables are 46.7%, 98.8%, 77.8%, 95.3%, and 94.5%, respectively.

**Table 3:** Significant multivariate predictors of *Aspergillus* versus non-fungal IE.

	Wald	β	p-value	Odds ratio	95% CI*
**Constant**	59.6	-5.82	<0.001	0.003	
**Absence of Fever**	7.5	1.76	0.006	5.79	1.65–20.31
**HAE**	31.5	3.89	<0.001	48.46	12.50–187.89
**PVE**	5.7	1.22	0.017	3.38	1.25–9.16
**Aortic abscess/ pseudoaneurysm**	6.8	1.45	0.009	4.26	1.43–12.68

*CI, confidence interval

To confirm the diagnostic criteria and diagnostic model, the AE group (to rule out those diagnosed based on GM alone) and the non-fungal endocarditis group (to rule out culture/serology negative cases) were compared by repeating the statistical analysis and retesting the diagnostic model. The total number of AE cases was 26 and the total number of non-fungal cases was 190.

Interestingly, fever was present in only 65.4% of the AE as compared to 93.7% of the non-fungal group (p < 0.001). There was a significant association between AE and acute limb ischemia at presentation (p = 0.05). HAE and PVE were the only significant risk factors associated with AE (p < 0.001 for each). Moreover, non-valvular and aortotomy site vegetations, as well as aortic abscess/pseudoaneurysm, were significantly associated with AE based on univariate analysis (p-value = 0.001, <0.001, and <0.001, respectively) (supporting data, S1 Table).

Significant univariate predictors of Aspergillus IE were entered in a stepwise logistic regression model with backward elimination. Details of the constructed regression model are shown in S2 Table of the supporting data. HAE was the most powerful predictor of AE, followed by abscess/pseudoaneurysm, PVE, then absence of fever.

The probability of a patient having AE accompanied by HAE, PVE, and abscess/pseudoaneurysm, but no fever, is 0.99. In contrast, the probability of a patient having AE accompanied by native valve IE, but no HAE and no abscess/pseudoaneurysm, is 0.001. Prediction of AE based on these four variables has a sensitivity, specificity, positive predictive value (PPV), negative predictive value (NPV), and overall diagnostic accuracy of 80.8%, 97.4%, 80.8%, 97.4%, and 95.4%, respectively.

### Treatment

All of the AE patients were indicated for cardiac surgery, and the majority underwent surgery (27 patients; 87.1%). In contrast, 259 (78.2%) non-fungal IE patients were indicated for surgery, but only 152 (45.9%) ultimately underwent surgery. The antifungal therapies used to treat the AE group are shown in Table 4; Eleven patients received dual antifungal therapy that included voriconazole. The high cost of echinocandins—available only within the last 4 years—limited their use in our center. All surviving patients were prescribed lifelong itraconazole except for two patients who were prescribed 1-year of voriconazole then were shifted to itraconazole, and one patient who received six months of voriconazole then was shifted to itraconazole.

**Table 4:** Antifungal treatment*.

Treatment	Number of patients
Antifungal preoperative	8
Antifungal combination	11
Amphotericin B	9
Liposomal Amphotericin B	2
Voriconazole	19
Echinocandin	2
Itraconazole (in-hospital)	1

*Data available for 24 patients.

### Patient outcomes

Patient outcomes are shown in Table 5. There was a trend (but not statistically significant) towards a higher incidence of severe sepsis and mortality in the *Aspergillus* group as compared to the non-fungal group (p = 0.098 and 0.097, respectively). There was no significant difference regarding embolization and severe heart failure. Thirteen AE patients died during hospitalization.

**Table 5:** Outcome in Aspergillus versus non-fungal IE patients.

Variable	Aspergillus	Non-fungal	p-value
Surgery performed	27 (87.1)	152 (45.9)	***< 0*.*001***
Any embolization (peripheral / pulmonary / mycotic aneurysms/splenic)	17 (54.8)	194 (58.6)	0.684
Severe sepsis	8 (27.6)	37 (14.1)	***0*.*098***
CHF FC III/IV	9 (33.3)	78 (29.7)	0.691
Mortality	13 (41.9)	92 (27.8)	***0*.*097***

Numbers are counts (column percentages).

The univariate predictors of mortality are shown in Table 6. PVE, the use of single versus dual antifungal agents, severe heart failure, and severe sepsis were significant predictors of mortality (p = 0.008, p = 0.012, p = 0.003, and p = 0.01, respectively).

**Table 6:** Univariate predictors of mortality in Aspergillus IE.

Variable	Mortality	No Mortality	p-value
Clinical characteristic			
Age	39.3 ±19.23	28.5 ±12.4	0.209
Male Gender	6 (46.2)	10 (55.6)	0.605
Underlying heart disease			
PVE	12 (92.3)	8 (44.4)	***0*.*008***
Rheumatic heart disease	2 (15.4)	6 (33.3)	0.412
Congenital heart disease	0	2 (11.1)	0.5
Normal heart	1 (7.7)	4 (22.2)	0.368
Echocardiographic characteristics			
Mitral IE	6 (46.2)	4 (22.2)	0.247
Aortic IE	4(30.8)	9 (50)	0.284
Abscess/pseudoaneurysm	4 (33.3)	9 (50)	0.367
EF	60.4 ±8.52	57.2 ±10.20	0.458
Treatment			
Anti-fungal combination	1 (10)	10 (66.7)	***0*.*012***
Anti-fungal use pre-operative	2 (20)	6 (46.2)	0.379
Surgery performed	10 (76.9)	17 (94.4)	0.284
Complications			
CHF FC III—IV	8 (66.7)	1 (6.7)	***0*.*003***
Severe sepsis	7 (53.8)	1 (6.3)	***0*.*01***
Embolization	8 (61.5)	9 (50)	0.524

Numbers are presented as mean ± SD or counts (column percentages).

## Discussion

FE is a disease of high mortality and morbidity [3, 4]. Delayed/mistaken diagnosis, long duration of symptoms before hospitalization, and extracardiac manifestations are common characteristics [2], a fact that contributes to delayed and wrong management in many cases [2]. AE is even worse [2, 5]; the disease lacks most of the clinical criteria used for diagnosis of IE; as blood culture is almost always negative [3, 4] and fever may be absent. Diagnosis is usually late and in many cases is made post-mortem [5]. While advances in antifungal treatments together with development of surgical techniques may represent a new era in the prognosis and fate of FE, many AE patients will not benefit from these advances due to late diagnosis [12,13].

Many reports have addressed the criteria used to diagnose invasive aspergillus infection. Invasive aspergillosis is typically diagnosed as being possible, probable, or proven [14–16]. Unfortunately, none of the criteria used in these reports addressed the diagnosis of AE.

The current study was conducted in a tertiary referral university hospital and included 374 patients with IE enrolled between February 2005 and June 2016. FE accounted for 43 cases and *Aspergillus* was the most common fungus (31 patients; 8.3% of the entire population group). Fever was present in 67.7% of AE patients as compared to 92.4% of the non-fungal group (p < 0.001). Acute limb ischemia at presentation was also significantly higher among AE patients (p = 0.014). HAE and PVE were the only significant risk factors associated with AE (p < 0.001 for each). Occurrence of AE on top of cardiac valve prostheses, cardiac devices, and following cardiac surgery was a common finding in many previous reports [1, 2, 11]. Moreover, mitral, non-valvular, and aortotomy site vegetations, as well as aortic abscess/pseudoaneurysm, were significantly associated with AE (p = 0.022, p = 0.004, p < 0.001, and p < 0.001, respectively).

Absence of fever (p = 0.006), HAE (p < 0.001), PVE (p = 0.017), and aortic abscess/pseudoaneurysm (p = 0.009) were associated with AE. The probability of a patient having AE along with HAE, PVE, and aortic abscess/pseudoaneurysm, but with no fever, was 0.92. In contrast, the probability of a patient having Aspergillus IE along with fever, native valve IE, but no HAE and no abscess/pseudoaneurysm, was 0.003. For predicting AE, the sensitivity, specificity, positive predictive value (PPV), negative predictive value (NPV), and overall diagnostic accuracy of these four variables were 46.7%, 98.8%, 77.8%, 95.3%, and 94.5%, respectively.

There was a trend towards a higher incidence of severe sepsis and mortality in the AE group (p = 0.098 and 0.097). Thirteen AE patients died during hospitalization. PVE, use of single versus dual antifungal agents, severe heart failure, and severe sepsis were significant predictors of mortality; (p = 0.008, p = 0.012, p = 0.003, and p = 0.01, respectively).

In our study, we used the Aspergillus Galactomannan Antigen to support diagnosis of AE; however, it is not commonly used to diagnose invasive aspergillosis in non-neutropenic patients. Galactomannan is a cell-wall constituent of *Aspergillus* that is released during fungal growth and has been widely used as a biomarker for early diagnosis of invasive aspergillus infection. Galactomannan molecules are cleared by neutrophils, which limits the diagnostic value of this test in non-neutropenic patients due to the likelihood of false negative results [17]. In one study of critically ill COPD patients (n = 90), two serum Aspergillus Galactomannan tests were performed on the first and fourth day after ICU admission. Positive and negative predictive values of invasive pulmonary aspergillosis of 89% and 85%, respectively, were observed [17]. In another study, serum Aspergillus Galactomannan was reported to be higher in patients with angio-invasive aspergillosis; which greatly resembles Aspergillus endocarditis, vs noninvasive airway invasive aspergillosis [18]. Serum Aspergillus Galactomannan antigen detection was used to diagnose cardiac aspergillosis in two nonneutropenic patients following surgery. The Galactomannan index was more than two-fold greater than the cut-off index of the kit, which decreased in response to surgical and antifungal therapies. Thus the Galactomannan index could prove to be a valuable approach for the diagnosis and follow-up of fungal endocarditis in such patients [19]. The Infectious Disease Society of America recommends the use of the Galactomannan as an useful adjunctive test for the early diagnosis of invasive aspergillosis in patients not receiving antifungal treatment [20].

While echinocandins are effective in treating candida FE [10, 21, 22], voriconazole has shown great superiority in treating invasive aspergillosis, including AE, and with greater tolerability by patients with minimal side effects [13, 23–25]. Some experts recommend antifungal combination therapy of voriconazole along with either echinocandins or amphotericin [3, 26, 27]. In the current study, combination antifungal therapy was strongly linked to decreased mortality (p = 0.012). In our study, voriconazole was an essential component of combination therapy.

## Conclusions

To our knowledge this is the first study to address diagnostic criteria for AE. Absence of fever, HAE, PVE, and aortic abscess/pseudoaneurysm were strong predictors of AE. Frequently, AE is diagnosed and treated late, leading to multiple morbidities and increased mortality. However, use of these criteria may lead to earlier diagnoses of AE and earlier treatment (e.g., voriconazole in combination with other antifungal agents), which will facilitate better patient outcomes.

## Limitations of the study

This study is limited by being a retrospective study comprised mainly of highly complicated patients as it was carried out in a tertiary referral center.

## Supporting information

S1 Table Clinical and echocardiographic characteristics of AE versus non-fungal IE groups after excluding AE cases with unavailable tissue diagnosis and non-AE cases with unknown microorganism. (DOCX)

S2 Table Significant multivariate predictors of Aspergillus versus non-fungal IE after excluding AE cases with unavailable tissue diagnosis and non-AE cases with unknown microorganism. (DOCX)
