# Correction: Aspergillus endocarditis: Diagnostic criteria and predictors of outcome, A retrospective cohort study

**DOI:** 10.1371/journal.pone.0203103

**Published:** 2018-08-23

**Authors:** Marwa Sayed Meshaal, Dina Labib, Karim Said, Mohammed Hosny, Mohammed Hassan, Said Abd Al Aziz, Amani Elkholy, Mervat Anani, Hussien Rizk

## Notice of Republication

An incorrect version of Fig 1 was published in error. This article was republished on August 10, 2018 to correct for this error. Please download this article again to view the correct version.
