# Correction: Aspergillus endocarditis: Diagnostic criteria and predictors of outcome, A retrospective cohort study

**DOI:** 10.1371/journal.pone.0208350

**Published:** 2018-11-27

**Authors:** Marwa Sayed Meshaal, Dina Labib, Karim Said, Mohammed Hosny, Mohammed Hassan, Said Abd Al Aziz, Amani Elkholy, Mervat Anani, Hussien Rizk

The published article [1] contains an incomplete ethics statement and the authors wish to correct this to the following:

Patients attending Kasr Al Ainy hospital sign a consent form at admission to agree to use of their clinical data for research. The authors were involved in patients' care and management during their clinical course, but data were de-identified when extracted from the clinical database for use in this study. The Cairo University Research Ethics Committee confirmed that this study was exempt from the requirement for approval. As per the University’s regulations, retrospective data analysis and other research that does not include interactions with or collection of identifying personal data from living human subjects, is exempt from Research Ethics Committee continuous review.
