# Supplementary material for: Aspergillus endocarditis: Diagnostic criteria and predictors of outcome, A retrospective cohort study
Source: PLoS One. 2018 Aug 9;13(8):e0201459. doi: 10.1371/journal.pone.0201459 (PMC6084895; doi:10.1371/journal.pone.0201459)
Supplement: S1 Table — (DOCX) [file pone.0201459.s001.docx]

**S1 Table. Clinical and echocardiographic characteristics of AE vs. non-fungal IE group***

| **Variable** | **Aspergillus**  **(n = 26)** | **Non fungal**  **(n = 190)** | **p-value** |
| --- | --- | --- | --- |
| **Clinical characteristics** |  |  |  |
| Age, years | 25 (19.5, 39) | 30 (24, 39) | 0.06 |
| Male gender | 11 (42.3) | 119 (62.6) | **0.05** |
| Symptom duration before referral,  days | 28 (13, 87) | 29 (14, 78) | 0.77 |
| Fever | 17 (65.4) | 178 (93.7) | **< 0.001** |
| Monoplegia / hemiplegia at  presentation | 3 (12) | 32 (16.8) | 0.77 |
| Acute limb ischemia at presentation | 4 (21.1) | 13 (6.8) | **0.05** |
|  |  |  |  |
| **Predisposing factors** |  |  |  |
| Health-care associated IE | 24 (92.3) | 31 (16.3) | **< 0.001** |
| IV drug abuse | 1 (3.8) | 25 (13.2) | 0.33 |
| Dialysis | 1 (3.8) | 13 (6.8) | 0.999 |
| Malignancy | 0 | 1 (0.5) | 0.999 |
| DM | 1 (4.3) | 13 (6.8) | 0.999 |
| Chronic steroid therapy / Collagen  disease | 1 (3.8) | 11 (5.8) | 0.999 |
| Prior IE | 0 | 7 (3.7) | 0.999 |
|  |  |  |  |
| **Underlying heart disease** |  |  |  |
| Normal heart | 4 (15.4) | 54 (28.4) | 0.16 |
| Rheumatic heart disease | 6 (23.1) | 68 (35.8) | 0.20 |
| Congenital heart disease | 2 (7.7) | 16 (8.4) | 0.999 |
| Prosthetic valve | 17 (65.4) | 37 (19.5) | **< 0.001** |
|  |  |  |  |
| **Echocardiographic characteristics** |  |  |  |
| Mitral vegetations | 9 (34.6) | 97 (51.1) | 0.12 |
| Aortic vegetations | 11 (42.3) | 63 (33.2) | 0.36 |
| Right-sided IE | 2 (7.7) | 42 (22.1) | 0.09 |
| Non-valvular vegetations | 6 (23.1) | 6 (3.2) | **0.001** |
| Aortotomy site vegetations | 10(52.6) | 0 | **< 0.001** |
| Aortic abscess / pseudoaneurysm | 11 (42.3) | 16 (8.4) | **< 0.001** |
| TTE diagnostic | 10 (52.6) | 120 (63.2) | 0.37 |
| EF, % | 59 (52, 62) | 63 (58, 68) | **0.02** |

Data are presented as n (column %) or median (interquartile range)
