# Supplementary material for: Aspergillus endocarditis: Diagnostic criteria and predictors of outcome, A retrospective cohort study
Source: PLoS One. 2018 Aug 9;13(8):e0201459. doi: 10.1371/journal.pone.0201459 (PMC6084895; doi:10.1371/journal.pone.0201459)
Supplement: S2 Table — (DOCX) [file pone.0201459.s002.docx]

**S2 Table. Significant multivariate predictors of Aspergillus versus non-fungal IE**

|  | **Wald** | **β** | **p-value** | **Odds ratio** | **95% CI** |
| --- | --- | --- | --- | --- | --- |
| **Constant** | 33.5 | -6.61 | <0.001 | 0.001 |  |
| **Absence of Fever** | 4.1 | 1.79 | 0.04 | 5.98 | 1.07-33.52 |
| **Healthcare-associated IE** | 22.4 | 4.78 | <0.001 | 119.28 | 16.50-862.15 |
| **Prosthetic valve IE** | 8.2 | 1.94 | 0.004 | 6.96 | 1.84-26.32 |
| **Aortic abscess/ pseudoaneurysm** | 10 | 2.81 | 0.002 | 16.52 | 2.91-93.67 |

CI, confidence interval
